# Socioeconomic Status Partially Mediates the Effects of Structural Racism on Youth Tobacco Use Initiation

**DOI:** 10.31586/gjeid.2024.1032

**Published:** 2024-08-17

**Authors:** Shervin Assari, Hossein Zare

**Affiliations:** 1 Department of Internal Medicine, Charles R. Drew University of Medicine and Science, Los Angeles, CA, United States; 2 Department of Family Medicine, Charles R. Drew University of Medicine and Science, Los Angeles, CA, United States; 3 Department of Urban Public Health, Charles R. Drew University of Medicine and Science, Los Angeles, CA, United States; 4 Marginalization-Related Diminished Returns (MDRs) Center, Los Angeles, CA, United States; 5 Department of Health Policy and Management, Johns Hopkins Bloomberg School of Public Health, Baltimore, MD, United States; 6 School of Business, University of Maryland Global Campus (UMGC), Adelphi, United States

**Keywords:** Racism, Tobacco Use, Smoking, Youth, Adolescents, Discrimination, Tobacco Susceptibility

## Abstract

**Background:**

Recent research has identified structural racism—systemic policies and practices that perpetuate racial inequalities—as a significant social determinant of population health. Studies utilizing data from the Adolescent Brain Cognitive Development (ABCD) study have shown an association between higher levels of state-level structural racism and increased tobacco use among youth in the United States. However, there has been limited exploration of the psychosocial mediators of this relationship, particularly in the context of youth aged 10-16 years.

**Objective:**

This study aimed to assess the roles of socioeconomic status (SES), tobacco susceptibility, and perceived discrimination as potential mediators in the relationship between state-level structural racism and youth tobacco initiation rates.

**Methods:**

We analyzed data from the ABCD study, a nationally representative longitudinal survey of 11,698 youth followed from ages 9/10 to 15/16. These data were combined with state-level indicators of structural racism. We employed structural equation modeling (SEM) to investigate the mediators of the association between structural racism and self-reported initiation of tobacco use, while controlling for individual and state-level covariates.

**Results:**

Our findings indicate that higher levels of structural racism were associated with increased rates of tobacco initiation among youth. This relationship was partially mediated by lower SES, but not by perceived discrimination or tobacco susceptibility.

**Conclusion:**

The association between structural racism and youth tobacco initiation appears to be influenced in part by the lower SES prevalent in states with higher levels of racism. These results highlight the need for addressing both racism and SES inequalities as key strategies for reducing tobacco disparities among youth.

## 1. Introduction

Tobacco use among youth remains a significant public health concern in the United States [1, 2], with substantial implications for long-term health outcomes [3]. The initiation of tobacco use during adolescence is particularly problematic, as it can lead to lifelong addiction, increased risk of chronic diseases, and premature mortality [4]. Despite extensive public health campaigns aimed at reducing tobacco use, recent data suggest that a significant proportion of youth still experiment with or regularly use tobacco products [5]. This issue is further compounded by socioeconomic status (SES) gap that may in part be a mechanism for the effects of racism on tobacco use in population groups [6].

Structural racism is increasingly recognized as a fundamental determinant of health, influencing a wide range of outcomes across different domains of life [7-12]. Structural racism refers to the systematic disadvantage of one racial group through policies, practices, and cultural norms that perpetuate inequalities in power, resources, and opportunities [8, 10, 11, 13-19]. This pervasive system of inequality operates at multiple levels, including state-level policies and practices that can shape the social and economic conditions in which people live [14, 20, 21]. Given that adolescence is a critical period for the development of health behaviors, understanding the role of structural racism in influencing these behaviors is crucial [22-26].

In the context of youth tobacco use, structural racism can manifest in various ways [27]. For instance, policies and practices that disproportionately affect minority communities, such as differential enforcement of tobacco regulations, targeted advertising in predominantly Black and Hispanic neighborhoods, and limited access to healthcare services, can create environments conducive to tobacco use initiation [28]. Furthermore, the stress and trauma associated with living in a racially stratified society can lead to coping mechanisms, such as smoking, as a form of stress relief [29, 30].

A recent study using data from the ABCD study have shown that state-level racism predicts tobacco use initiation among youth, with those in more racist states being more likely to begin using tobacco (Assari and Zare, Under Review). This finding builds on previous research by Weissman, Hatzenbuehler, McLaughlin, and others, which explored the effects of structural stigma on youth mental health, particularly in marginalized groups such as lesbian gay bisexual (LGB), Black, Latinx, and female youth [31-33]. One study found that LGB and Latinx youth, and females, experienced higher internalizing and externalizing symptoms in states with high structural stigma, while another study linked lower family income to reduced hippocampal volume and higher internalizing psychopathology, effects mitigated in states with more generous anti-poverty policies [31]. In addition, they found that LGB youth residing in states with high levels of structural stigma experienced elevated internalizing and externalizing symptoms compared to those in states with lower levels of stigma. In states with lower structural stigma, there were no significant differences in externalizing symptoms between LGB and heterosexual youth. Similarly, Latinx youth and females in high structural stigma states exhibited higher levels of externalizing symptoms compared to their counterparts in low stigma states. However, structural stigma related to race did not show a significant association with internalizing or externalizing symptoms among Black youth. That study provided new evidence that macro-level social environments, characterized by structural stigma, contribute to negative mental health outcomes for marginalized youth. This structural stigma partially explains the observed disparities in externalizing symptoms across different groups [32]. Finally, structural stigma was associated with smaller hippocampal volumes in Black and Latinx youth, suggesting that macro-level social environments significantly impact the mental and neurodevelopmental health of marginalized youth [33].

Although the existing literature has documented various health disparities linked to structural racism, including differential access to healthcare, variations in disease prevalence, and disparities in health behaviors [16, 19, 34, 35], research specifically examining the mechanism by which state-level structural racism and youth tobacco initiation are linked is limited. While some studies have explored the impact of community-level factors on youth smoking [36, 37], these studies do not account for SES, perceived discrimination, and perceived susceptibility as potential mechanisms for the broader structural and systemic influences of racism on youth tobacco use [38-41]. Thus, there is a clear need for studies that explore mediators of the effects of structural racism on tobacco use.

To understand the relationship between structural racism and youth tobacco use, it is essential to draw on relevant theoretical frameworks. Fundamental Cause theory considers racism as a root cause of health disparities. Bruce Link and colleagues posit that racism, as a fundamental cause, systematically influences a wide range of health outcomes by affecting access to essential resources, including economic opportunities, educational attainment, and healthcare [7, 42]. Structural racism operates through institutional mechanisms, such as discriminatory policies and practices, that perpetuate inequality and constrain opportunities for minority groups [16, 19, 43]. These mechanisms are resilient, adapting to different social and economic contexts to maintain racial disparities [34, 35]. In the context of tobacco use, structural racism can shape the environment in which youth grow up, influencing their exposure to risk factors and access to protective resources [44]. For instance, segregation and social stratification, rooted in red lining and discriminatory housing and banking policies have concentrate minority populations in neighborhoods with high levels of tobacco advertising and low access to preventive health services [45]. This theoretical framework highlights the necessity of addressing the systemic nature of racism to understand and mitigate its impact on health behaviors, including the initiation of tobacco use among youth.

The Social Ecological Model [46] provides a comprehensive lens through which to examine how multiple levels of influence, from individual to societal, shape health behaviors. This model posits that individual behaviors are influenced by interactions with their environment, including family, community, and broader societal structures [47]. In the case of tobacco use, individual choices are not made in isolation but are affected by the availability of tobacco products, cultural norms, and policies that regulate tobacco use [48].

Life Course Theory [49] further enriches this perspective by emphasizing the importance of timing and context in shaping health trajectories. This theory conceptualizes adolescence as a vulnerability stage that can shape future habits, behaviors, and lifestyle [50]. This theory suggests that exposures and experiences during critical developmental periods, such as adolescence, can have lasting effects on health outcomes [51]. Applying Life Course Theory to the study of structural racism and tobacco use initiation highlights how early experiences of racial discrimination and socioeconomic disadvantage can set the stage for later health behaviors and outcomes [51, 52].

Although the existing literature has documented various health disparities linked to structural racism, including differential access to healthcare, variations in disease prevalence, and disparities in health behaviors [16, 19, 34, 35], research specifically examining the association between state-level structural racism and youth tobacco initiation is limited. While some studies have explored the impact of community-level factors on youth smoking [36, 37], they often do not account for the broader structural and systemic influences that shape these environments. Moreover, much of the existing research has focused on adult populations, leaving a gap in understanding how structural racism affects younger individuals, particularly during the critical period of early adolescence [38-41]. There is a clear need for studies that consider the complex interplay between structural racism and youth health behaviors across different states. Such research can provide valuable insights into the mechanisms by which structural racism influences the initiation of tobacco use among youth and highlight the role of state-level policies and practices in perpetuating these disparities.

The purpose of this study is to investigate the association between state-level structural racism and the subsequent initiation of tobacco use among Adolescent Brain Cognitive Development (ABCD) youth aged 10-16 years. Specifically, this study aims to assess the relationship between state-level indicators of structural racism (e.g., racial disparities in incarceration rates, education, and economic opportunities) and youth tobacco initiation rates. The result of this study has significant implications for public health policy and intervention strategies. By identifying the structural factors that contribute to youth tobacco initiation, we can develop more targeted and effective prevention programs. Addressing structural racism as a determinant of health is crucial for reducing disparities and promoting equity in health outcomes. This research will contribute to the growing body of knowledge on the social determinants of health, particularly in the context of youth behaviors, and underscore the importance of policy-level interventions in addressing public health challenges.

## 2. Methods

### 2.1. Design, Sample, and Sampling

We performed a secondary analysis of data from the Adolescent Brain Cognitive Development (ABCD) study [53-58], a national longitudinal investigation focused on a racially and socioeconomically diverse cohort of pre-adolescent children as they transition into adolescence. Participants were primarily recruited from schools, and further details about the study's aims, methodology, and measures can be found in existing literature. The ABCD dataset is characterized by a broad representation across race, SES, and geographical regions. Our analysis utilized a sample consisting of 22,538 observations from 11,878 children [53-58].

### 2.2. Analytical sample

Analytical sample included in our analysis was 11,698. Eligibility criteria for this study was being tobacco naïve at baseline. No other factor was considered as inclusion or exclusion criteria, so participants could enter our analysis regardless of their race/ethnicity, SES background, or residence.

### 2.3. Predictor (Structural Racism)

The measure included 31 items reflecting state-level anti-Black racism. These items captured explicit racial attitudes and prejudices, as collected from individual responses to Project Implicit (2002–2017), the General Social Survey (1973–2014), and the American National Election Survey (1992–2016). The items covered various aspects of race, including attitudes toward Black individuals, endorsement of racial stereotypes, and perceptions of the prevalence and impact of racial discrimination [31-33].

### 2.4. Outcome (Tobacco Use Initiation)

Tobacco use in this study was assessed every six months, employing instruments such as the web-based Timeline Follow-Back, which covers various substances including tobacco. Tobacco use initiation was defined as the first instance of using nicotine (regardless of the product) beyond a mere puff [59].

### 2.5. Mediators

#### Socioeconomic Status (SES):

Our SES variable was constructed as a principal component derived from parental education, household income, financial difficulties, and family structure. This measure was continuous, with higher scores indicating higher SES. Parental education levels were ascertained by asking, "What is the highest grade or level of school you have completed or the highest degree you have received?" and similarly for their partners. The highest educational attainment of either parent was used as the parental education variable. Levels of this variable were as below: less than high school, High school graduate, some college/associate degree, college graduate, master’s degree, and doctoral degree. Family income was a continuous measure with the following levels, based on the response to the question: "What is your total combined family income for the past 12 months? This should include income (before taxes and deductions) from all sources, such as wages, rent, social security, disability and veteran’s benefits, unemployment benefits, worker’s compensation." Levels of family income were less than 5K, 5-12k, 12-16k, 16-25k, 25-35k, 35-50k, 50-75k, 75-100k, 100-200k, and >=200k. For financial difficulties, participants were asked the following seven questions: “In the past 12 months, has there been a time when you and your immediate family experienced any of the following:” (1) “Needed food but could not afford to buy it or could not afford to go out to get it?”, (2) “Were without telephone service because you could not afford it?” (3) “Did not pay the full amount of the rent or mortgage because you could not afford it?”, (4) “Were evicted from your home for not paying the rent or mortgage?”, (5) “Had services turned off by the gas or electric company, or the oil company would not deliver oil because payments were not made?”, (6) “Had someone who needed to see a doctor or go to the hospital but did not go because you could not afford it?”, and (7) “Had someone who needed a dentist but could not go because you could not afford it?” Responses to each of these items were either 0 or 1. We calculated a mean score with a potential range between 0 and 1—a higher score indicating higher financial difficulties. This variable was a continuous measure [60]. Parents also reported their marital status, which was used to dichotomize family structure into married (two-parent cohabitation) and other statuses.

#### Perceived Discrimination:

Perceived discrimination was assessed using a set of seven items administered at the end of the one-year follow-up period. One example of these items is: "How often do the following people treat you unfairly or negatively because of your ethnic background?" Responses were recorded on a scale from 1 to 5, where 1 indicated "almost never" and 5 indicated "very often." Participants had the option to respond with "don't know" or to refuse to answer. The overall score was calculated as the average of the items, resulting in a range from 1 to 5, with higher scores reflecting greater perceived discrimination.

#### Tobacco Susceptibility:

Tobacco susceptibility was assessed at baseline using three items that asked participants (youth) about their curiosity, openness to future use, and expectations regarding tobacco use if offered by a friend. This variable was treated as a continuous measure, with higher scores indicating greater cognitive and perceived susceptibility to tobacco use.

### 2.6. Covariates

The control variables in our study included demographic factors such as race/ethnicity, age, and assigned sex at birth. Parents reported their child's date of birth, allowing us to calculate age in months as a continuous variable. Assigned sex was coded dichotomously (male and female). The child's race/ethnicity, as reported by parents, was the moderator variable, with categories including non-Latino White (used as the reference category), African American/Black, Asian, Latino, American Indian/ Native Hawaiian/Pacific Islander, Other, and unknown.

### 2.7. Data Analysis

For statistical analysis, we utilized Stata 18.0. We conducted univariate analyses, reporting means and standard deviations (SD), as well as frequencies/percentages overall. For bivariate analysis, we ran Pearson correlation. For multivariable analyses, we employed structural equation model (SEM) in the pooled sample. The primary outcome was any tobacco use over the follow up period (more than a puff). The predictor was state-level racism, treated as a continuous measure. We controlled for potential confounders, including age, sex-assigned at birth, and race/ethnicity. Mediators included tobacco susceptibility, SES, and perceived discrimination. All of these mediators were continuous measures. SES variable was a latent factor composed of parental education, household income, and financial difficulties. We ensured there was no multicollinearity among the variables, as indicated by all correlations being weaker than 0.4. Results were presented as standardized coefficient, 95% confidence intervals (CI) and p-values.

### 2.8. Ethics

The ethics approval for the ABCD study was initially granted by the University of California, San Diego (UCSD) Institutional Review Board (IRB). Informed assent was obtained from all participating children, and parental consent was secured. Our secondary analysis was exempt from full IRB review.

## 3. Results

Table 1 shows descriptive data of the participants. As this table shows, participants were between 9- and 10-year-old at baseline. 52% of participants were non-Latino White and 52% were male (assigned sex at baseline). Overall, 3.5% of the participants-initiated tobacco use (more than a puff).

As shown by Table 2, tobacco use was positively and significantly correlated with structural racism.

**Figure 1.**
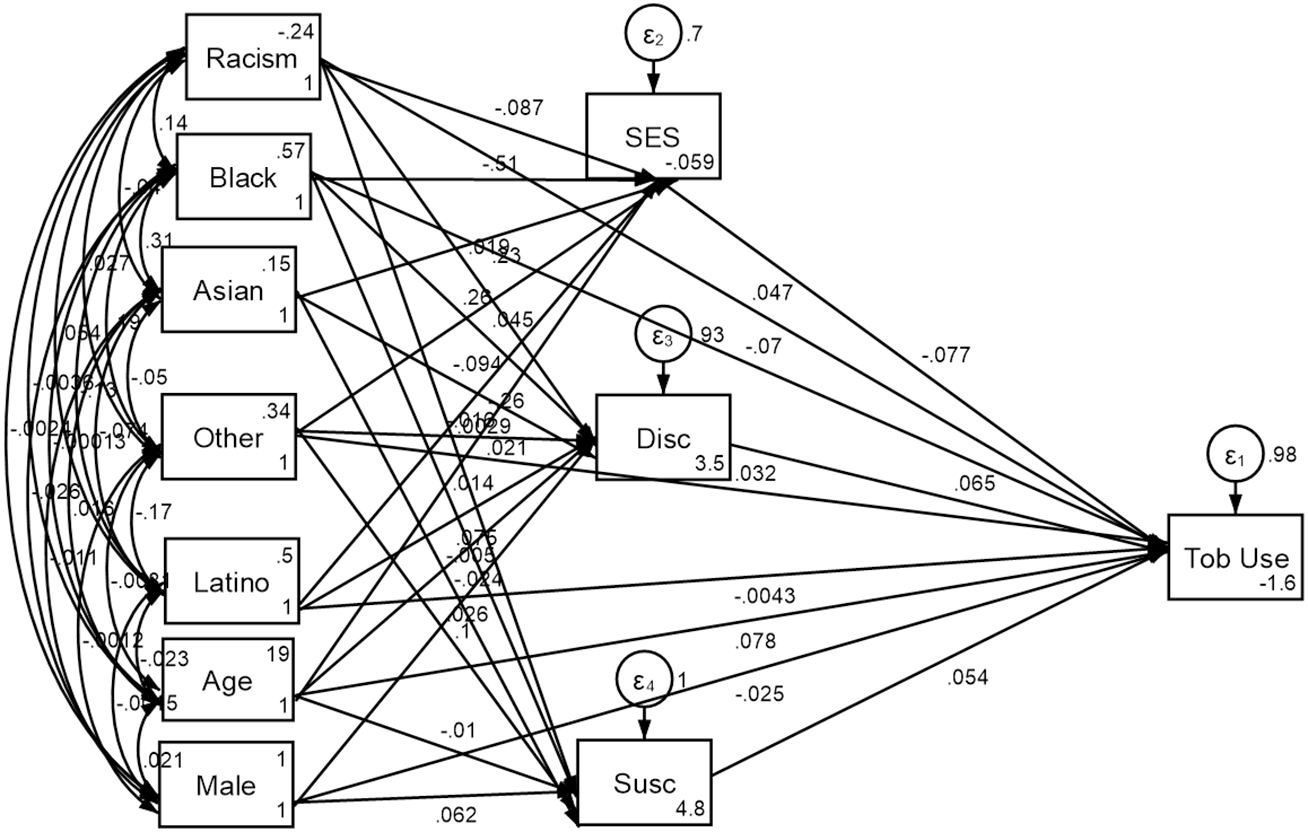
Summary of structural equation model

Table 3 and Figure 1 show that higher levels of structural racism are associated with increased rates of tobacco initiation among youth. This association remained significant even after accounting for demographic and socioeconomic factors such as age, sex, and race/ethnicity. Additionally, a pathway was identified from higher structural racism to lower SES, which then led to subsequent tobacco use, suggesting that SES partially mediates the effect of structural racism on subsequent tobacco initiation. However, this mediating effect was not observed for the other two potential mediators—tobacco susceptibility and perceived discrimination. Therefore, neither tobacco susceptibility nor perceived discrimination mediated the impact of structural racism on subsequent tobacco use.

## 4. Discussion

The study's findings underscore a significant association between state-level structural racism and the initiation of tobacco use among youth aged 10-16. Our analysis revealed that state level of structural racism is predictive of higher rates of youth tobacco initiation. This association was mediated by lower SES but not tobacco susceptibility or perceived discrimination.

To understand the relationship between structural racism and youth tobacco use, it is essential to draw on relevant theoretical frameworks. Fundamental Cause theory considers racism as a root cause of health disparities. Bruce Link and colleagues posit that racism, as a fundamental cause, systematically influences a wide range of health outcomes by affecting access to essential resources, including economic opportunities, educational attainment, and healthcare [7, 42]. Structural racism operates through institutional mechanisms, such as discriminatory policies and practices, that perpetuate inequality and constrain opportunities for minority groups [16, 19, 43]. These mechanisms are resilient, adapting to different social and economic contexts to maintain racial disparities [34, 35]. In the context of tobacco use, structural racism can shape the environment in which youth grow up, influencing their exposure to risk factors and access to protective resources [44]. For instance, segregation and social stratification, rooted in red lining and discriminatory housing and banking policies have concentrate minority populations in neighborhoods with high levels of tobacco advertising and low access to preventive health services [45]. This theoretical framework highlights the necessity of addressing the systemic nature of racism to understand and mitigate its impact on health behaviors, including the initiation of tobacco use among youth.

The Social Ecological Model [46] provides a comprehensive lens through which to examine how multiple levels of influence, from individual to societal, shape health behaviors. This model posits that individual behaviors are influenced by interactions with their environment, including family, community, and broader societal structures [47]. In the case of tobacco use, individual choices are not made in isolation but are affected by the availability of tobacco products, cultural norms, and policies that regulate tobacco use [48].

Life Course Theory [49] further enriches this perspective by emphasizing the importance of timing and context in shaping health trajectories. This theory conceptualizes adolescence as a vulnerability stage that can shape future habits, behaviors, and lifestyle [50]. This theory suggests that exposures and experiences during critical developmental periods, such as adolescence, can have lasting effects on health outcomes [51]. Applying Life Course Theory to the study of structural racism and tobacco use initiation highlights how early experiences of racial discrimination and socioeconomic disadvantage can set the stage for later health behaviors and outcomes [51, 52].

There exists a literature that highlights the adverse health effects of structural racism [16, 17, 19, 34, 35, 43]. Prior studies have documented the link between structural racism and various health outcomes, including mental health issues, chronic diseases, and substance use [20, 42, 61]. However, our study adds to this body of work by expanding this literature to youth tobacco use initiation and by utilizing state-level measure of racism. Combined with the literature, our result provides critical insights into the role of structural racism in shaping health behaviors at a formative stage of life.

Several mechanisms may explain the observed relationship between structural racism and youth tobacco initiation. First, structural racism may be associated with less effective tobacco control policies [62, 63]. States with high levels of structural racism often have policies that limit economic and educational opportunities for minority populations [64]. These exposures may lead to increased stress and psychological distress among youth [65, 66]. Racism-related stress can manifest in coping behaviors, such as tobacco use, especially in environments where tobacco products are readily accessible [67-70].

Additionally, targeted marketing of tobacco products in states with higher racism may involve minority communities and expose them to advertisement that can exacerbate the risk of initiation [71-73]. Tobacco companies have historically targeted Black and Hispanic communities with advertisements for menthol cigarettes and other tobacco products, exploiting social and economic vulnerabilities. The normalization of smoking within these communities, reinforced by targeted marketing and limited access to cessation resources, creates a permissive environment for youth tobacco use.

Furthermore, the cumulative impact of structural racism, including experiences of discrimination and marginalization, can lead to diminished expectations for the future among minority youth. This outlook may reduce the perceived risks associated with tobacco use and increase the likelihood of experimentation and regular use.

Racism is blamed as a mechanism that reduces the reurns of SES on brain development, economic wellbeing, and tobacco use, and health. Now we found that SES also reduces SES through racist policies that block opportunities. Due to these mechanisms, we may observe tobacco use and substance use of high SES and also low SES racialized groups and minoritized groups such as Black, Asian, and Hispanic youth and adults on substances such as tobacco, alcohol, and marijuana.

Exposure to stress related to low SES impacts youth, which may lead them to seek coping mechanisms such as tobacco use. Trauma may create a state of psychological and emotional distress for which youth may turn to tobacco use.

Systemic racism significantly hampers economic and educational opportunities for minoritized populations through multiple mechanisms. Discriminatory practices in the labor market, such as hiring biases and wage disparities, restrict access to well-paying jobs and career advancement for people of color. These practices often result in lower incomes and limited financial stability. Moreover, systemic racism in education manifests in the form of underfunded schools, racially biased disciplinary actions, and limited access to advanced courses, all of which contribute to lower educational attainment and reduced opportunities for higher education. The disparities in educational resources and quality further exacerbate the gap in economic mobility, as individuals from marginalized backgrounds are less likely to gain the qualifications needed for high-paying careers. Additionally, systemic racism influences housing policies, such as redlining and discriminatory lending practices, which segregate communities and limit access to quality education and economic opportunities. These structural barriers reinforce a cycle of poverty, restricting the capacity of minoritized populations to achieve economic and educational mobility. As a result, systemic racism not only directly impacts income and employment opportunities but also perpetuates long-term disparities in wealth accumulation and social mobility.

Family SES is often protective against substance use in youth because it typically provides a stable and supportive environment that promotes healthy behaviors. Families with higher SES generally have better access to resources, such as quality education, healthcare, and extracurricular activities, which can serve as protective factors against substance use. These families often have greater knowledge about the risks associated with substance use and can provide their children with the necessary information and guidance to make informed decisions. Additionally, higher SES families are more likely to reside in safer neighborhoods with lower availability of drugs and alcohol, reducing the likelihood of exposure. Conversely, youth in low SES environments are more exposed to risk factors for substance use, including higher rates of stress due to financial instability, less parental supervision due to parents working multiple jobs, and greater exposure to environments where substance use is normalized. The availability of substances in low SES areas can be higher due to the presence of more liquor stores or illegal drug markets. Peer influence also plays a significant role, as youth in low SES environments may be more likely to associate with peers who engage in substance use, thereby increasing their own risk. The combination of limited knowledge, greater availability of substances, and peer influence creates a challenging environment for low SES youth, making them more vulnerable to substance use.

In the face of such distress, some youth may turn to tobacco and other substances as a way to alleviate their emotional pain and manage the stress associated with their experiences. Tobacco and substance use can offer a temporary escape from the harsh realities of discrimination, providing momentary relief from feelings of inadequacy or social exclusion. However, this coping mechanism can quickly become maladaptive, leading to the development of addictive behaviors that compound the initial stress. The cycle of using substances to manage stress exacerbates the underlying issues and hinders the development of healthier coping strategies. Understanding this dynamic is crucial for developing effective interventions and support systems that address both the psychological impact of discrimination and the prevention of substance use among affected youth.

### 4.1. Policy Implications

The findings of this study have significant implications for economic policies and tobacco control strategies. To address youth tobacco use disparities in states with high levels of racism, it is crucial to close the socioeconomic status (SES) gap and enhance the SES of affected populations. Measures such as increasing access to education, raising the minimum wage, adjusting tax policies, or providing direct financial assistance could help mitigate tobacco disparities linked to racism. Policies aimed at reducing racial disparities by addressing SES inequalities may prove effective. By narrowing the gaps in education, income, and employment, we can potentially reduce the impact of racism on youth tobacco use, particularly in vulnerable families. Even if structural racism cannot be entirely eliminated, reducing its economic impact on families may help prevent tobacco use among youth. Examples of such policies include increasing cash assistance, raising wages, enhancing parents' employability, and implementing tax policies that benefit low-SES individuals.

### 4.2. Limitations

This study has a few limitations. The short-term follow-up period resulted in only 3.5% of the youth population initiating tobacco use, limiting our ability to thoroughly investigate mediators and moderators of tobacco initiation. Additionally, we did not examine factors such as tobacco attitudes, peer risk, and parental involvement. The measurement of structural racism is inherently complex, and the measure we use may not fully capture its multifaceted nature. Our variable only measured racism at the state level, overlooking variations in racism at the community and school levels. Despite these limitations, this study is the first to explore mediators of the association between structural racism and youth tobacco initiation.

### 4.3. Future Research Directions

Future research should investigate additional potential mediators and moderators in the relationship between structural racism and youth tobacco use. Specifically, studies should explore the roles of parental control, monitoring, internalizing symptoms, and school performance as mechanisms through which racism exerts its effects. Future research should test if feelings of anxiety, depression, and feelings of helplessness may connect racism to tobacco use. Additionally, examining the effectiveness of targeted policy interventions in disrupting the impact of structural racism on youth tobacco use is crucial. It is also important to study the intersectionality of structural racism with other social determinants of health, such as gender, socioeconomic status, and immigration status. Furthermore, research should delve into the neurological mechanisms underlying these observed pathways.

## 5. Conclusion

In summary, our study found that low SES, rather than higher perceived discrimination or tobacco susceptibility, largely accounts for the significant association between state-level structural racism and youth tobacco use initiation. While factors such as peer risk, neighborhood environments, education policies, and the density of tobacco retail outlets may also contribute, low SES appears to be a key explanatory factor. To effectively reduce youth tobacco use linked to structural racism, policymakers should focus on addressing the economic disparities perpetuated by racism.

## Figures and Tables

**Table 1.** Descriptive data of the overall participants (n = 11,698).

	Mean	SE
**Age**	9.48	0.00
State-Level Racism	−0.18	0.01
Parental Education at Baseline (1-6)	3.91	0.01
Household Income at Baseline (1-11)	7.23	0.02
Financial Difficulties at Baseline (Mean)	0.07	0.00
Perceived Discrimination	1.18	0.00
Tobacco Susceptibility/	1.08	0.00
Socioeconomic Status (Principal Component)	0.13	0.02
	n	%
**Sex**		
Female	5,628	48.00
Male	6,098	52.00
**Race/Ethnicity**		
Asian	628	5.35
AIAN/ Native Hawaiian /Pacific Islander	342	2.91
Non-Hispanic Black	2,126	18.1
Hispanic White	2,394	20.38
Other	98	0.83
Non-Hispanic White	6,109	52
Unknown	52	0.44
**Household Marital Status (Baseline)**		
Unmarried	7,916	67.49
Married	3,813	32.51
**Tobacco Use (Subsequent)**		
No	11,329	96.85
Yes	369	3.15

American Indian and Alaska Native: AIAN

**Table 2.** Correlation between state-level structural racism and other study variables.

	1	2	3	4	5	6	7	8	9	10	11	12	13	14	15
1 Structural Racism	1.00														
2 Race (Black)	0.15*	1.00													
3 Race (Latino)	0.06*	−0.17*	1.00												
4 Race (Asian)	−0.03*	−0.05*	−0.07*	1.00											
5 Race (Other)	0.03*	−0.13*	−0.16*	−0.05*	1.00										
6 Sex (Male)	0.00	−0.02*	0.00	0.00	−0.01	1.00									
7 Age	−0.01	0.01	−0.02*	0.02*	−0.01	0.02*	1.00								
8 Financial Difficulties at Baseline	0.12*	0.22*	0.06*	−0.05*	0.06*	0.01	−0.01	1.00							
9 Married Household at Baseline	−0.11*	−0.34*	−0.10*	0.06*	−0.04*	0.02*	0.01	−0.27*	1.00						
10 Household Income at Baseline	−0.19*	−0.37*	−0.20*	0.07*	−0.03*	0.00	0.04*	−0.43*	0.54*	1.00					
11 Parental Education at Baseline	−0.14*	−0.26*	−0.25*	0.10*	0.00	0.00	0.01	−0.31*	0.39*	0.64*	1.00				
12 Tobacco Susceptibility at Baseline	0.01	0.02	0.00	−0.01	0.03*	0.06*	−0.01	0.02*	−0.02*	−0.03*	−0.01	1.00			
13 Perceived Discrimination	0.08*	0.18*	0.05*	−0.02*	0.02*	0.08*	−0.02*	0.15*	−0.13*	−0.20*	−0.17*	0.07*	1.00		
14 Socioeconomic Status (Principal Component)	−0.19*	−0.39*	−0.21*	0.09*	−0.04*	0.00	0.02*	−0.62*	0.72*	0.88*	0.75*	−0.02*	−0.21*	1.00	
15 Subsequent Tobacco Use	0.05*	0.00	0.03*	−0.03*	0.01	0.00	0.06*	0.05*	−0.07*	−0.06*	−0.07*	0.06*	0.05*	−0.08*	1.00

* p < 0.05; Pearson correlation

**Table 3.** Association between state-level racism and subsequent youth tobacco initiation via socioeconomic status, perceived discrimination, and tobacco susceptibility.

	Standardized Coefficient	SE	95%	CI	p
Subsequent Tobacco Use					
Structural Racism	0.05	0.01	0.03	0.06	< 0.001
Socioeconomic Status (SES)	−0.08	0.01	−0.10	−0.05	< 0.001
Perceived Discrimination	0.07	0.01	0.04	0.09	< 0.001
Tobacco Susceptibility	0.05	0.01	0.03	0.07	< 0.001
Age	0.08	0.01	0.06	0.10	< 0.001
Race/Ethnicity (Black)	−0.07	0.01	−0.09	−0.05	< 0.001
Race/Ethnicity (Latino)	0.00	0.01	−0.02	0.02	0.664
Race/Ethnicity (Other)	0.03	0.01	0.01	0.05	0.001
Sex (Male)	−0.03	0.01	−0.04	−0.01	0.006
Intercept	−1.63	0.18	−1.98	−1.27	< 0.001
					
Socioeconomic Status (SES)					
Structural Racism	−0.09	0.01	−0.10	−0.07	< 0.001
Age	0.02	0.01	0.01	0.04	0.010
Race/Ethnicity (Asian)	0.23	0.07	0.09	0.38	0.002
Race/Ethnicity (Black)	−0.51	0.02	−0.56	−0.46	< 0.001
Race/Ethnicity (Latino)	−0.26	0.01	−0.28	−0.24	< 0.001
Race/Ethnicity (Other)	0.04	0.01	0.03	0.06	< 0.001
Intercept	−0.06	0.15	−0.36	0.24	0.697
					
Perceived Discrimination					
Structural Racism	0.02	0.01	−0.01	0.05	0.194
Age	−0.02	0.01	−0.05	0.00	0.081
Race/Ethnicity (Asian)	−0.09	0.04	−0.17	−0.02	0.019
Race/Ethnicity (Other)	−0.02	0.02	−0.05	0.01	0.290
Race/Ethnicity (Black)	0.26	0.02	0.22	0.30	< 0.001
Race/Ethnicity (Latino)	0.08	0.01	0.05	0.10	< 0.001
Sex (Male)	0.10	0.01	0.07	0.13	< 0.001
Intercept	3.53	0.27	3.01	4.05	< 0.001
					
Tobacco Susceptibility					
Structural Racism	0.00	0.01	−0.02	0.02	0.762
Age	−0.01	0.01	−0.03	0.01	0.294
Race/Ethnicity (Asian)	0.00	0.01	−0.03	0.02	0.633
Race/Ethnicity (Other)	0.03	0.01	0.01	0.04	0.009
Race/Ethnicity (Black)	0.01	0.01	−0.01	0.04	0.215
Male	0.06	0.01	0.04	0.08	< 0.001
Intercept	4.80	0.18	4.44	5.15	< 0.001
